# Vorinostat protects against calcium oxalate-induced kidney injury in mice

**DOI:** 10.3892/mmr.2015.3964

**Published:** 2015-06-19

**Authors:** LI WANG, WEI CHEN, ZHONGJIANG PENG, CHANGCHENG LIU, CAIHONG ZHANG, ZHIYONG GUO

**Affiliations:** 1 Department of Nephrology, Changhai Hospital, Second Military Medical University, Shanghai 200433, P.R. China; 2 Department of Cell Biology, Division of Basic Medicine, Second Military Medical University, Shanghai 200433, P.R. China

**Keywords:** calcium oxalate, histone deacetylase, kidney injury, oxidative stress

## Abstract

The present study aimed to examine the effect of the histone deacetylase inhibitor, vorinostat (SAHA), on renal function in a calcium oxalate crystal mouse model, and to investigate the mechanism underlying the renoprotective effect of SAHA. Calcium oxalate crystal formation was induced in 8 week-old male C57BL/6 mice by administering 100 mg/kg glyoxylate for 7 days. A total of 24 male C57BL/6 mice were randomly divided into a control group and the following experimental groups: 50 mg/kg normal saline + 100 mg/kg glyoxylate; 50 mg/kg dimethyl sulfoxide (DMSO) + 100 mg/kg glyoxylate; and 50 mg/kg SAHA + 100 mg/kg glyoxylate. The mice in each of the experimental groups were injected with the saline, DMSO or SAHA into their abdominal cavities 6 h prior to the glyoxylate injection. The mice were sacrificed after 7 days, following which blood and urine samples were collected. The kidneys were harvested to analyze the levels of calcium concentrations and the levels of malondialdehyde (MDA), superoxide dismutase and glutathione reductase. Immunohistochemical staining and semi-quantitative analyses were performed to detect the expression levels of osteopontin (OPN) and CD44. Renal tubular cell apoptosis was detectedusing a TUNEL assay. The concentrations of calcium and malondialdehyde were significantly decreased in the SAHA group, and calcium oxalate crystals in the kidney tissue and the expression levels of OPN and CD44 in the SAHA group were lower, compared with the other experimental groups. SAHA significantly reduced the urinary excretion of KIM-1 and renal tubular cell apoptosis. In conclusion, SAHA reduced calcium oxalate crystal deposition and protected against kidney injury.

## Introduction

Kidney stone disease is major health concern and an economic burden on health systems (1). Kidney stones, which can cause kidney injury, are common and frequent problems. A previous study revealed that kidney stones are associated with a significant loss of kidney function and contribute to the risk of end-stage kidney disease (2). Shoag *et al* (3) suggested that a history of kidney stones is associated with an increased risk of chronic kidney disease (CKD) and requirement for dialysis treatment among females, when adjusted for co-morbidities.

Although several forms of surgery, including extracorporeal shock wave lithotripsy, are performed to remove kidney stones, stone recurrence and kidney injury remain serious issues. Therefore, preventing and treating kidney stones is critical. Calcium oxalate stones remain the most common type of nephrolithiasis, accounting for ~80% of kidney stones (4). The exact mechanism of kidney stone formation remains to be fully elucidated. Currently, investigations are focusing on crystal formation in the kidney, which is the early stage of stone development. Reactive oxygen species (ROS) are considered to be important in kidney crystal formation. Huang *et al* (5) reported that ROS activation of nicotinamide adenine dinucleotide phosphate (NADPH) contribute to renal tubular cell injury, however, the inhibitor of NADPH decreases the expression of kidney injury molecular-1 (KIM-1). In addition, other previous studies have revealed that oxalate-mediated oxidative stress in erythrocytes may contribute to the tubular damage and stone accumulation in patients with hyperoxaluria (6).

Several previous studies have suggested epigenetic modulation in kidney diseases. Inhibition of histone deacetylase (HDAC) activity attenuates renal injury via anti-inflammatory and anti-fibrotic effects (7,8). Advani *et al* (9) demonstrated that long-term administration of vorinostat, also termed suberanilohydroxamic acid (SAHA) attenuates renal injury ina mouse model of diabetes by reducing oxidative nitrosative stress (9). The transcription factor, Nrf2, is a key regulator of antioxidant-responsive genes and is bound to its inhibitor, Kelch-like ECH-associated protein 1 (Keap1). Wang *et al* (10) demonstrated that the HDAC inhibitor, trichostatin A (TSA), suppresses the expression of Keap1, activates Nrf2 and enhances Nrf2-ARE binding. Furthermore, TSA contributes to neuroprotection following cerebral ischemia, the effects of which are not observed in Nrf2-deficient mice (10). Taken together, these findings suggest that HDAC inhibitors exert renoprotective and anti-oxidative functions, and may be a potential drug target for kidney stones. According to previous experimental methods (11), the renal calcium oxalate animal model was established in the present study to investigate whether SAHA improves kidney injury and to determine the possible mechanism of kidney stone development.

## Materials and methods

### Animals

A total of 24 healthy wild type male C57BL/6 mice, aged 8 weeks, were obtained from the Shanghai SLAC Lab Animal Co, Ltd. (Shanghai, China) and were raised in the SPF Animal Facility of the Second Military Medical University (Shanghai, China). All animals had free access to standard laboratory food and drinking water, and were maintained under a controlled 12 h light/day cycle at 20–25°C with a relative humidity of 55–65%. Animal care and procedures followed the recommendations of the NIH Guide for the Care and Use of Laboratory Animals. The present study was approved by the Committee on Ethics of Biomedicine Research, Second Military Medical University.

### Drugs and chemicals

Vorinostat was purchased from Selleck Chemicals (Houston,. TX, USA) and was dissolved in DMSO (Sigma-Aldrich, St. Louis, MO, USA) to a final concentration of 50 mg/2 ml. Glyoxylate was purchased from Tokyo Chemical Industry Co., Ltd. (Tokyo, Japan), the Oxidative Stress kit was purchased from Jiancheng Institute of Biotechnology (Nanjing, China) and the von Kossa Staining Commercial kit was purchased from Shanghai Shunbai Biotechnology, Co., Ltd. (Shanghai, China). A mouse monoclonal antibody against osteopontin (OPN; cat. no. sc-73631; 1:50) and the appropriate secondary antibody were purchased from Santa-Cruz Biotechnology, Inc. (Santa Cruz, CA, USA), and a rabbit polyclonal antibody against CD44 (cat. no. 15675-1-AP; 1:100) was purchased from Proteintech Group (Chicago, IL, USA). The TUNEL commercial kit was purchased from EMD Millipore (Billerica, MA, USA).

### Study design and procedure

A total of 24 mice were acclimatized for 1 week prior to the experiments. The animals were assigned randomly into four groups (n=6 per group): Control group (no treatment); saline group, (intraperitoneal injection of 50 mg/kg/day saline); DMSO group (an intra-abdominal injection of 50 mg/kg/day DMSO); and SAHA group (intraperitoneal injection of 50 mg/kg/day SAHA). Following treatment for 6 h, all groups, with the exception of the control group, received an intraperitoneal injection of 100 mg/kg/day glyoxylate. The procedures were performed daily for 7 days.

### Biochemical indicators

The mice were placed in metabolic cages for 24 h for urine collection. The blood was collected from all animals prior to sacrifice under 3% pentobarbital sodium anesthesia. The levels of serum creatinine (Scr) and blood urea nitrogen (BUN) were measured with the assitance of the University of Shanghai Hospital Clinical Laboratory (Shanghai, China) using a BC-2800 Vet Animal Auto Biochemistry Analyzer (Guangzhou Shihai Medical Equipment Co., Ltd., Guangdong, China). The ratios of urinary concentration of calcium to creatinine were also calculated with the assistance of the University of Shanghai Hospital Clinical Laboratory using a BC-2800 Vet Animal Auto Biochemistry Analyzer (Guangzhou Shihai Medical Equipment Co., Ltd.).

### Measurement of urinary KIM-1

The protein levels of KIM-1 were quantified using a modified enzyme-linked immunosorbent assay (ELISA) system. The wells of an ELISA plate (USCN Life Science Inc., Wuhan, China) were coated with 200 µl anti-KIM-1 polyclonal antibody (1.0 *µ*g/ml) and incubated overnight at 4°C in 50 mM carbonate solution. The wells were blocked with a 1% bovine serum albumin solution in phosphate-buffered saline (PBS) and were washed four times with PBS containing 0.05% Tween-20 (PBST). The serum samples (100 *µ*l) and urine samples (200 *µ*l) were then added to each well at room temperature for 3 h. Following incubation, the samples were washed four times with PBST prior to incubation with biotinylated anti-KIM-1 monoclonal antibody. Following incubation with primary antibody, the samples were incubated with horseradish peroxidase-conjugated streptavidin, with tetramethylbenzidine as the substrate. The levels were quantified using a microplate reader to detect the concentration and a standard curve was created.

### Kidney sample collection and pathological analysis

All animals underwent heart perfusion, and the left kidneys were removed and placed into eppendorf tubes with 4% paraformaldehyde. Heart perfusion enables the preservation of the kidneys for morphological observations. The kidneys were embedded in paraffin wax (Hubei Laike Medicine Instrument Co., Ltd., Xiangfan, China) and cross-sections were sliced at 3 *µ*m (Leica RM2235; Hubei Lai Ke Medicine Equipment Co., Ltd.). These slices were then prepared for von Kossa immunohistochemistry and TUNEL staining. The right kidneys were stored in eppendorf tubes in a refrigerator at −80°C for determining the calcium oxalate concentration and levels of malondialdehyde (MDA), superoxide dismutase (SOD) and glutathione reductase (GSH). The slices underwent deparrafinization and hydration using a series of dilutions of xylene and alcohol, followed by staining using the von Kossa kit and subsequent eosin counterstaining (Beyotime Institute of Biotechnology, Haimen, China). The stained slices were then assessed using microscopy (Nikon Eclipse 50i; Shanghai Henghao Instruments Co. Ltd., Shanghai, China) for the distribution of calcium oxalate crystals, characterized by black calcium oxalate crystal deposits. The number of crystals in a total cross-sectional tissue area was determined using Adobe Photoshop software version 7.0 (Adobe Systems, Inc., San Jose, CA, USA) in 20 randomly selected fields (magnification, x200). For determination of the levels of OPN and CD44,paraffin sections were high pressure treated for 2 min and blocked with 0.5% H_2_O_2_ in methanol for 15 min, washed in 0.01 M PBST and further treated with non-fat milk in PBS for 30 min at room temperature. The slides were then incubated overnight at 4°C with primary antibodies against OPN and CD44, followed by incubation with the secondary antibody. The positive staining of OPN and CD44 were measured as the ratio of integral optical density/field of kidney cross-sections, using Image-pro Plus software version 6.0. Using the TUNEL kit and methyl green counterstaining, the number of TUNEL-positive cells were counted using Image-Pro Plus software. A total of six randomly-selected fields were used under a magnification of x400.

### Statistical analysis

Statistical analysis was performed using SPSS 17.0 software (SPSS, Inc., Chicago., IL, USA). The data are expressed as the mean ± standard deviation. Statistical significance was determined using one-way analysis of variance and Student's t-test. P<0.05 was considered to indicate a statistically significant difference.

## Results

### SAHA ameliorates kidney injury induced by CaOx and reduces crystal formation

**Figure 1:**
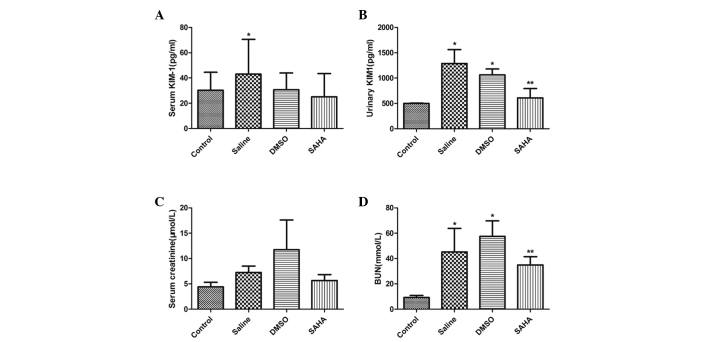
(A) Serum levels of KIM-1 in mice (*P<0.05, compared with control). (B) Urinary levels of KIM-1 (*P<0.05, compared with the control; **P<0.05, compared with the saline and DMSO groups). Levels of (C) serum creatinine and (D) BUN were determined (*P<0.05, compared with the control; **P<0.05 compared with the saline and DMSO groups). All data are expressed as the mean ± standard deviation. BUN, blood urea nitrogen; DMSO, dimethyl sulfoxide; SAHA, vorinostat group; KIM-1, kidney injury molecular-1.

The results of the ELISA demonstrated a significant increase in the urinary and serum levels of KIM-1 in the saline and DMSO-treated groups. The SAHA group exhibited a significant reduction in urinary KIM-1 excretion (Fig. 1A and B). The levels of BUN and Scr were higher in the saline and DMSO groups, compared with the control group. The level of BUN in the SAHA group decreased significantly, however, this group exhibited no significant change in levels serum creatinine (Fig. 1C and D).

**Figure 2:**
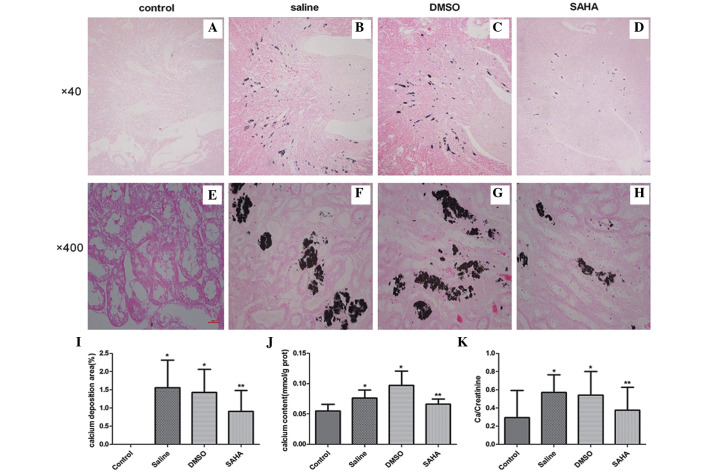
(A–H) von Kossa staining and eosin counter staining of the mouse kidney. Calcium oxalate crystal deposits were observed in the tubules of the corticomedullary junction area. The crystals were significantly reduced in the SAHA group. (I) Renal calcium deposition was determined by semi-quantitative analysis, by the area of positive staining from 20 randomly selected fields (magnification x200). ^*^P<0.001, compared with the control; ^**^P<0.05 compared with the saline and DMSO groups. (J) Renal calcium content (^*^P<0.05, compared with the control; ^**^P<0.05, compared with the saline and DMSO groups). (K) Ratio of the urinary concentration of Ca/creatinine (^*^P<0.05, compared with the control; ^**^P<0.05, compared with the saline and DMSO groups). All data are expressed as the mean ± standard deviation. DMSO, dimethyl sulfoxide; SAHA, vorinostat group.

Following intraperitoneal injection of glyoxylate for 7 days, the deposition of calcium oxalate crystals was assessed using microscopy (Nikon Eclipse 50i) in the experimental groups, in the region between the renal cortex and the medulla, particularly in the corticomedullary junction. Treatment with SAHA ameliorated crystal aggregation (Fig. 2A–H). The semi-quantitative assessment of kidney crystal formation revealed that the numbers of crystals in the saline and DMSO groups were significantly higher, compared with that in the control group (P<0.001). However, the SAHA-treated kidneys exhibited fewer crystals (P<0.05; Fig. 2I; Table I) than these two groups. In addition, the calcium concentrations in the kidney tissues of the SAHA group were significantly lower, compared with the other experimental groups (Fig. 2J). The presence of crystals led to a rise in urinary concentrations of Ca/creatinine, as observed in the saline and DMSO groups. The Ca/creatinine concentrations were significantly decreased following treatment with SAHA (Fig. 2K). No significant difference was observed between the DMSO and saline groups.

### SAHA decreases the level of CaOx-induced oxidative stress

**Figure 3:**
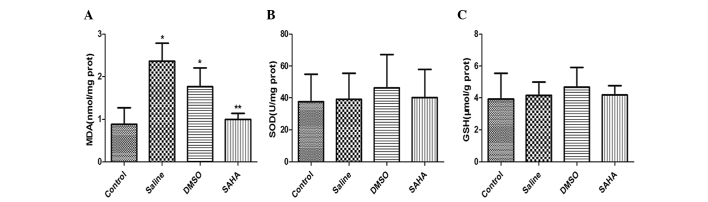
Right kidney contents. (A) The content of MDA in the right kidney (^*^P<0.05, compared with the control group; ^**^P<0.05, compared with the saline and DMSO groups). (B) The content of SOD in the right kidney and (C) the content of GSH in the right kidney. All values are expressed as the mean ± standard deviation. DMSO, dimethyl sulfoxide; SAHA, vorinostat; MDA, malondialdehyde; SOD, superoxide dismutase; GSH, glutathione reductase.

To determine whether the level of oxidative stress was associated with SAHA treatment, the concentrations of MDA, SOD and GSH in the kidney tissues were assessed. The results indicated that glyoxylate caused a significant increase in the concentrations of MDA in the saline and DMSO groups, compared with the control. By contrast, the MDA content inthe SAHA-treated group was significantly lower than in the saline and DMSO groups (Fig. 3A). Although SAHA did not significantly alter the levels of SOD (Fig. 3B) or GSH (Fig. 3C) in the kidney tissues, a marginal increase was observed.

### SAHA alters the expression levels of OPN and CD44, and reduces apoptosis induced by CaOx

**Figure 4:**
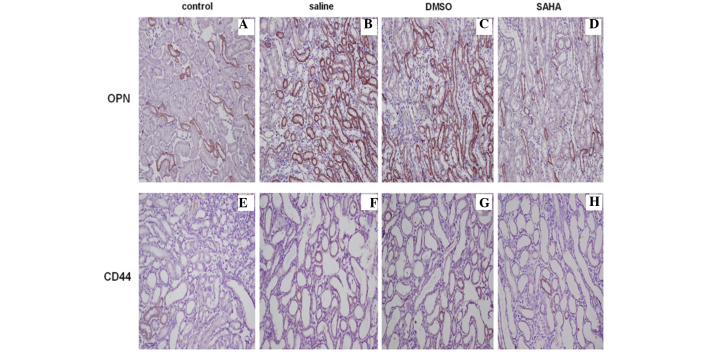
Immunostaining of OPN and CD44 in the rat kidney tissues. Immunostaining for (A–D) OPN and (E–H) CD44 in tissue samples from rat kidneys. The staining for OPN and CD44 were markedly decreased in the SAHA group, compared with the DMSO and saline groups (magnification, x200). OPN, osteopontin; SAHA, vorinostat.

Immunohistochemical staining of the kidney tissues revealed that the expression of OPN located in the kidney tubules was upregulated in the saline and DMSO groups, particularly at the corticomedullary border region. The expression of CD44 was observed in a diffuse localization pattern in normal rat kidney and was weaker, compared with the expression of OPN. Kidney crystal formation resulted in high levels of expression in the proximal tubular cells at the corticomedullary junction area, however, the SAHA group exhibited markedly lower expression levels of OPN and CD44, confirmed by semi-quantification (Fig. 4A–H; Table I).

**Figure 5:**
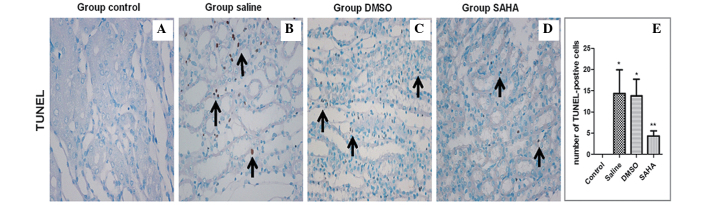
TUNEL staining to determine the apoptotic response of the tissue samples. (A–D) Representative images of TUNEL stained rat kidneys. The number of TUNEL-positive cells was significantly lower in the SAHA group, compared with the DMSO and saline groups. The black arrows indicate TUNEL-positive cells (magnification, x400). (E) Quantification of the number of TUNEL-positive cells using semi-quantitative analysis of the area of positive staining from eight randomly selected fields. The data are expressed as the mean ± standard deviation (^*^P<0.001, compared with the control; ^**^P<0.05, compared with the saline and DMSO groups. DMSO, dimethyl sulfoxide; SAHA, vorinostat group.

The results of the TUNEL staining revealed stained nuclei in the area from the cortex to medulla in the saline and DMSO groups, particularly in the corticomedullary junction. The SAHA group exhibited weaker staining. The number of TUNEL-positive cells in the SAHA group was significantly lower, compared with those in the saline and DMSO groups (Fig. 5A–D), which was similar to the data obtained in the semi-quantification analysis (Fig. 5E).

## Discussion

Nephrolithiasis is one of the primary causes of obstructive nephropathy and lower quality of life. Thus, efforts are required to identify novel methods to prevent kidney stone formation and improve renal function. The present study assessed the efficacy of treatment with SAHA as a prophylactic agent for kidney injury induced by CaOx. Serum and urine biochemical analyses indicated a significant reduction in the level of BUN and in the urinary concentration ratio of Ca/creatinine in the SAHA-treated group, compared with the other treatment groups. KIM-1 is highly specific and sensitive in identifying acute kidney injury (12). The urinary levels of KIM-1 increased in the saline and DMSO groups, however, Scr was unchanged. These results suggested that an increase in KIM-1 may assist in the diagnosis of kidney injury at anearlier stage, compared with increases in Scr. Han *et al* (13) reported a similar finding. In addition, data from the present study indicated that SAHA reduced the urinary excretion of KIM-1, which may account for SAHA protection against CaOx-induced kidney injury.

Based on the results of von Kossa staining and calcium detection in the present study, SAHA administration reduced renal calcium deposition, consistent with the marked anti-nephrolithic effect of SAHA. Cho *et al* reported that the two predominant stone components are calcium oxalate (71.5%) and uric acid (15.3%). In addition, metabolic syndrome has also been associated with a significantly increased risk of uric acid calculi development, particularly in patients with impaired fasting glucose and hyper-triglyceridemia (14). The possibility that SAHA reduces calcium deposition by changing the metabolic state cannot be excluded. In an investigation of Japanese males, inflammation was suggested as a possible underlying mechanism of the association between obesity and kidney stone formation (15). SAHA also exerts an anti-inflammatory effect. Advani *et al* (9) demonstrated that SAHA decreases the expression of eNOS in the kidneys of diabetic mice and in cultured endothelial cells. Mice genetically deficient in eNOS, however, are resistant to the attenuating effects of SAHA (9). Previous animal studies have indicated that ROS have been recognized as a vital mediator, which can lead to oxidative stress during crystal formation (16). Hirose *et al* (17) revealed that renal tubular cell injury, particularly injury caused by mitochondrial damage and oxidative stress, can induce the early stage of calcium oxalate crystal formation in mice (17). Khan *et al* (18) revealed that hydroxyl-l-proline contributed to stone formation, along with gene expression of macromolecular modulators (MMs) in hyperoxaluric rats. Treatment with the NADPH oxidase inhibitor, apocynin, however, resulted in a nearly complete absence of crystals and changes of MMs. Therefore, CaOx crystallization is likely regulated by ROS, which is produced, in part, through the activation of NADPH oxidase (18). The present study revealed that rats treated with SAHA exhibited decreased levels of MDA and increased levels of SOD and GSH, suggesting that treatment with SAHA reduced calcium oxalate crystal formation and was associated with the decrease in ROS production, demonstrating an anti-oxidative effect. However, no significant difference was observed in the levels of SOD and GSH. The reason may be that SAHA decreases ROS production more than it increases antioxidant enzymes.

Calcium oxalate crystal adherence to injured tubular epithelial cell results in stone aggregation, and several types of proteins are involved in the pathological process. Kanlaya *et al* detected a total of 14 proteins as differentially expressed proteins, with western blot analysis suggesting that oxalate induced the upregulation of α-enolase and immunoblotting analysis indicating that the downregulation of RhoA was associated with the identified proteins (19). OPN is a type of glycoprotein that is widely expressed in several tissues, and is involved in several physiological and pathological processes, including cell adhesion, migration, signaling, inflammation and biomineralization (20). A previous report demonstrated that OPN is expressed predominantly in the distal convoluted tubule and collecting duct, and the results of immunohischemical staining and *in situ* molecular hybridization have supported this finding (21). A minor difference was that Ullrich *et al* revealed that OPN is expressed indistal and proximal tubules (22). Okada *et al* identified OPN to be present in the tubules and stones, and the expression of OPN is upregulated during kidney injury (11). The present study observed that the distribution of OPN is similar to that of crystal retention, which is predominantly located in the tubules at the corticomedullary junction area. Asselman *et al* reported the same distribution (23). These data indicated that OPN co-localized with CaOx crystals. Tsuji *et al* reported increased renal expression of OPN in hyperoxaluric rats and a significant reduction in expression following transfection with OPN siRNA (24). The increased expression of OPN may be caused by ROS production, derived from cell injury, and inhibiting oxidative stress may reverse this. As expected, SAHA downregulated the expression of OPN in the present study.

CD44 is a ubiquitous transmembrane glycoprotein and serves as a cell surface receptor for hyaluronic acid and OPN, the biological activities of which primarily depends on their interaction with CD44. The expression of CD44 is rare in normal kidneys, however, it is increased in kidney tissues with renal crystals. Asselman *et al* revealed that CD44 is expressed at the luminal surface of crystal-binding renal tubular cells, however, it was not expressed in cells lacking affinity for crystals (23). In a metabolic syndrome mouse model, Fujii *et al* demonstrated that renal crystallization contributes to upregulation of the expression levels of OPN and CD44 (25). The present study also demonstrated that OPN and CD44 were expressed at sites where crystals were retained. Treatment with SAHA resulted in a reduction in the expression levels of OPN and CD44.

In unilateral ureteral obstruction model mice, Pang *et al* demonstrated that Trichostatin A inhibits caspase-3 phosphorylation and ameliorates tubular epithelial cell apoptosis (26). In HK-2 cells, Khaskhali *et al* demonstrated that exposure to high levels of Ca or CaOx crystals causes injury to renal epithelial cells, although the two do not work synergistically. High levels of Ca induce cell injury and may be caused by the production of ROS, and the development of oxidative stress (27). Niimi *et al* (28) observed mitochondrial collapse within renal tubular cells and cell apoptosis was assessed using cleaved caspase-3. In the present study, TUNEL staining indicated that kidney crystals led to cell apoptosis and that treatment with SAHA improved apoptosis. These data suggested that HDAC inhibitors act in a renoprotective manner against apoptosis.

In conclusion, SAHA may be an effective prophylactic agent against CaOx by reducing kidney injury and promoting mechanisms involved in renoprotective functions, at least in part, by decreasing oxidative stress and cell apoptosis, and down-regulating the expression levels of OPN and CD44. Therefore, HDAC inhibitors may have therapeutic potential for the treatment of kidney stones, particularly in refractory and recurrent nephrolithiasis. However, further investigations are required to clarify the specific mechanism underlying this response.

## Figures and Tables

**Table I:** Semi-quantification of calcium oxalate crystal formation and immunohistochemical staining for CD44 and OPN.

Group	Crystal deposition (%)	Average CD44 (IOD)	Average OPN (IOD)
Control	0	0.05±0.01	0.42±0.04
Saline	1.56±0.76^a^	0.09±0.03^a^	0.49±0.07^a^
DMSO	1.43±0.63^a^	0.08±0.01^a^	0.49±0.06^a^
SAHA	0.91±0.58^b^	0.05±0.02^b^	0.43±0.05^b^

The data are expressed as the mean ± standard deviation

a P<0.05, vs. control;

b P<0.05, vs. saline and DMSO). DMSO, dimethyl sulfoxide; IOD, integrated optical density.

## Abbreviations

ESRD: end-stage renal disease
CKD: chronic kidney disease
SAHA: suberoylanilide hydroxamic acid
DMSO: dimethyl sulfoxide
MDA: malondialdehyde
SOD: superoxide dismutase
GSH: glutathione reductase
KIM-1: kidney injury molecular-1
HDAC: histone deacetylase
TSA: trichostatin A
CaOx: calcium oxalate
Scr: serum creatinine
BUN: blood urea nitrogen
OPN: osteopontin
HA: hyaluronan
ROS: reactive oxygen species
MMs: macromolecular modulators
NADPH: nicotinamide adenine dinucleotide phosphate
