# Vorinostat protects against calcium oxalate-induced kidney injury in mice

**DOI:** 10.3892/mmr.2020.11782

**Published:** 2020-12-14

**Authors:** Li Wang, Wei Chen, Zhongjiang Peng, Changcheng Liu, Caihong Zhang, Zhiyong Guo

Mol Med Rep 12: 4291-4297, 2015; DOI: 10.3892/mmr.2015.3964

Subsequently to the publication of the above paper, an interested reader has drawn to the authors' attention that, in Fig. 4, there appeared to be an overlap between the data shown in the 4B (OPN/saline) and the 4C (OPN/DMSO) panels, such that these data may have been derived from the same original source even though different experimental conditions were described in the Figure; furthermore, the Fig. 4F (saline) and 4G (DMSO) panels appeared to be very similar, as if they were tissue sections from the same specimen.

Upon examining the final proofs of this article, the authors have determined that the immunohistochemical images of the saline groups were incorrectly selected. A further examination of the original data files disclosed that an error had been made in the labelling of some of the original images.

The corrected version of Fig. 4, including the correct data for Fig. 4B and F (for the immunostaining of OPN and CD44, respectively, under saline conditions), is shown opposite. Note that these errors did not affect either the results or the conclusions reported in this paper, and all the authors agree to this Corrigendum. The authors are grateful to the Editor of *Molecular Medicine Reports* for allowing them the opportunity to publish this Corrigendum, and apologize to the readership for any inconvenience caused.

## Figures and Tables

**Figure 4.**
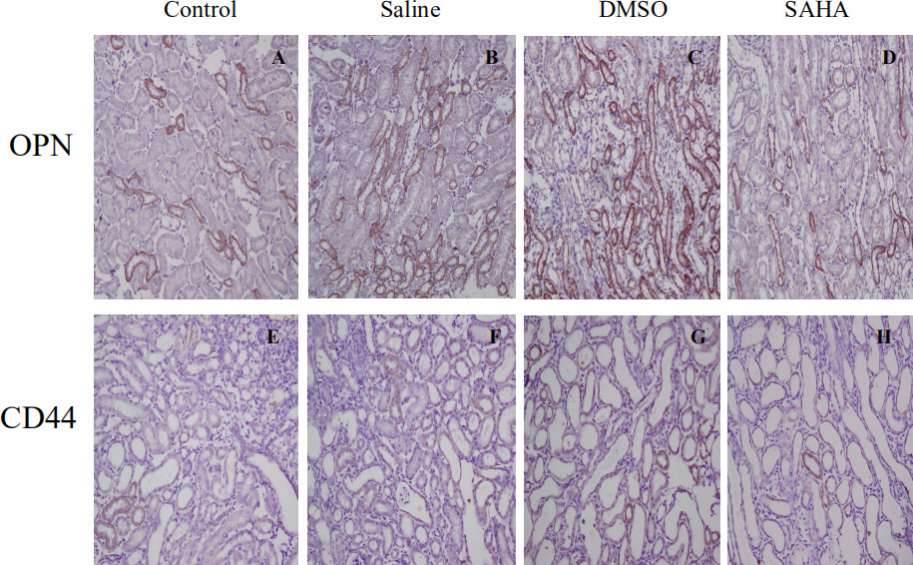
Immunostaining of OPN and CD44 in the rat kidney tissues. Immunostaining for (A-D) OPN and (E-H) CD44 in tissue samples from rat kidneys. The staining for OPN and CD44 were markedly decreased in the SAHA group, compared with the DMSO and saline groups (magnification, ×200). OPN, osteopontin; SAHA, vorinostat..

